# Through an animal’s eye: the implications of diverse sensory systems in scientific experimentation

**DOI:** 10.1098/rspb.2024.0022

**Published:** 2024-07-17

**Authors:** Joanna S. Brebner, Maria Loconsole, Daniel Hanley, Vera Vasas

**Affiliations:** ^1^ Research Centre on Animal Cognition (CRCA), Centre for Integrative Biology (CBI); CNRS, University Paul Sabatier – Toulouse III, Toulouse, France; ^2^ School of Biological and Behavioural Sciences, Queen Mary University of London, London, UK; ^3^ Department of General Psychology, University of Padova, Padova, Italy; ^4^ Department of Biology, George Mason University, Fairfax, VA, USA; ^5^ School of Life Sciences, University of Sussex, Brighton BN1 9RH, UK

**Keywords:** animal vision, anthropocentric bias, behavioural experiments, experiment design, sensory biology

## Abstract

‘Accounting for the sensory abilities of animals is critical in experimental design.’ No researcher would disagree with this statement, yet it is often the case that we inadvertently fall for anthropocentric biases and use ourselves as the reference point. This paper discusses the risks of adopting an anthropocentric view when working with non-human animals, and the unintended consequences this has on our experimental designs and results. To this aim, we provide general examples of anthropocentric bias from different fields of animal research, with a particular focus on animal cognition and behaviour, and lay out the potential consequences of adopting a human-based perspective. Knowledge of the sensory abilities, both in terms of similarities to humans and peculiarities of the investigated species, is crucial to ensure solid conclusions. A more careful consideration of the diverse sensory systems of animals would improve many scientific fields and enhance animal welfare in the laboratory.

## 1. Introduction

**Figure 1.**
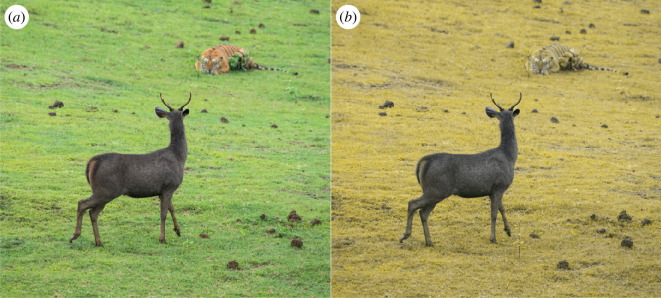
A tiger crouching in the grass, as seen by human observers (*a*), appears very different for dichromatic animals without a separate red channel—such as their ungulate prey (*b*). To illustrate the available dichromatic information, we simulated the view of a person with protanopia-type colour blindness (lacking a red channel) using the ‘Daltonize’ plugin of ImageJ [14]. The original image (*a*) was taken by Vraghur and shared under the Creative Commons Attribution-ShareAlike 4.0 International licence (https://creativecommons.org/licenses/by-sa/4.0/deed.en).

Anthropocentric biases influence the ways we design behavioural experiments. The perceptual abilities of an animal will determine how it approaches behavioural challenges, and even subtle limitations in experimental design can lead to hidden flaws. Despite repeated calls to consider the animal’s perspective [1–8], as humans, we are biased by our own experiences of the world and have a strong tendency to assume similar sensory abilities (or a lack thereof) in the animals that surround us [9,10]. The result is unaccountable biases and flaws. Famously, fighting bulls were once thought to be ‘provoked’ by red capes; of course, bulls do not perceive red colours, and they react to the matador’s challenge for entirely different reasons [11,12]. Similarly, while tigers might burn bright for William Blake, they are grass-coloured to their ungulate prey [13] (figure 1).

The scientific world is not free from such biases. When designing experiments, we inadvertently make anthropocentric assumptions, forgetting that our senses are the result of our unique evolutionary history. Our human view of the ‘real world’ is specific to us; in the same way any species’ perception of the ‘real world’ is specific to them. Here, we focus on the unique challenges posed by the diversity of sensory systems found in research animals. In the following sections, we highlight the importance of assessing and reporting sensory cues (§2); the challenges posed by novel set-ups (§3); the benefits of designing experiments based on a species’ evolutionarily relevant sensory modalities (§4); the dangers of well-known assumptions (§5) and finally, the considerations necessary for a species’ lifecycle and ecology (§6). We argue for the need to question traditionally accepted methods in light of our knowledge of biology and sensory ecology. To this end, we highlight best practice guidelines to minimize the risk of common pitfalls (table 1), and illustrate our perspective with several tangible examples where common practices may be flawed.

**Table 1.** Checklist of recommended considerations when designing experiments with animals.

**sensory biology and ethology**		
S1	take into consideration the entire sensory system of your animal	Which sensory modalities does your experimental species possess? On which sense(s) does your animal primarily rely? How is the sensory information used by the animal?
S2	choose a behavioural response that is within the natural repertoire of your animal	Can the animal provide a natural response to the test? Is the animal required to acquire a new behaviour? In which contexts do you expect to find this behavioural response?
**environment**		
E1	environment allowing for the full expression of the behaviours of your animal	Do they have all the necessary resources for expressing their normal behaviours (e.g. bedding material, shelter)?
E2	(if in the laboratory) provide the appropriate cage or housing systems that account for species-specific needs	Are you providing the subject(s) with a sufficiently large space? Are you working on a social species or a solitary species? Do you account for the consequences of social deprivation on your task?
E3	account for the natural biological cycle of your animal	Is your animal nocturnal or diurnal? Do you have an artificial light/dark cycle? Do you account for behavioural differences related to the circadian rhythm of that species (e.g. hours of maximum or minimum activity)? Do you account for seasonal/age-related variations (e.g. hormones, food availability)?
**methods**		
M1	carefully check your experimental set-up and paradigm	Does your paradigm or setting account for possible confounds? Do you have someone else’s feedback? If you are using electronic tools (e.g. monitors), are the stimuli perceived as intended?
M2	look for alternative explanations for the behaviour of interest	Can the behavioural response be triggered by factors other than the experimental manipulation? Can similar behaviours be mistaken for the behaviour of interest?
M3	account for interindividual variability	Can variance in your results be ascribed to individual differences (e.g. levels of boldness, neophobia)? Do you know the average individual variability expected within the measured behaviour in your species?

## 2. Assess and report all sensory cues: the tale of the bees, the artificial flowers and the colours of quinine

**Figure 2.**
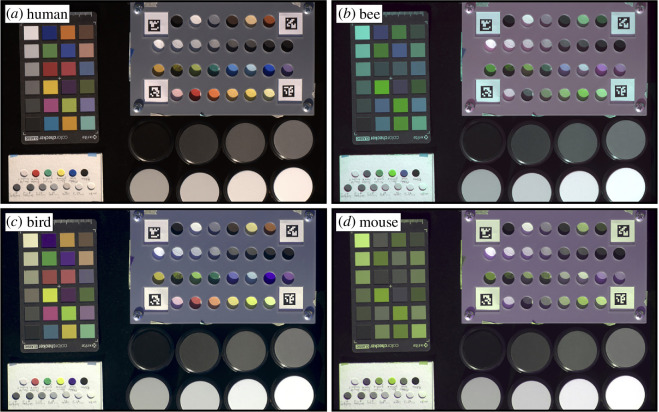
Colours appear very differently to humans (*a*) than to bees (*b*), birds (*c*) or mice (*d*). Note that there is no direct correspondence between human- and animal-perceived colours. The false-coloured images were made using Vasas *et al*. [17]. For the bees, the red, green and blue channels of the image correspond to the ultraviolet, green and blue photoreceptor responses, respectively; for birds, the red, green and blue channels correspond to the red, green, and the mean of the ultraviolet and blue photoreceptor responses, respectively; for mice, the red, green and blue channels correspond to the mean of the ultraviolet and green, green and ultraviolet photoreceptor responses, respectively. A gamma correction of 0.5 was applied to the images.

We will begin with discussing experiments relying on animal vision, a comparatively well understood sensory modality (presumably owing to its importance to us) [15,16]. Eye morphologies and colour vision abilities (figure 2) of non-human animals rarely match our own [18–20], with the exception of great apes [21]. A straightforward example of this is the perception of ultraviolet light, which is of importance to many animals, such as most insect pollinators, birds, reptiles and fish [22–25]. Although this is a widely understood fact, the ultraviolet dimension of vision is often deliberately excluded, or worse, left unmeasured and unreported in behavioural experiments [22,26–28] under the assumption that this signal will not affect the subject’s behaviour. Unfortunately, this omission is rarely supported by evidence, and ultraviolet colours can have a large effect on individual behaviour (e.g. mate selection [29,30], foraging choice [31], camouflage [28]; S1, E1, table 1). In some cases, accounting for ultraviolet colours can fundamentally change our interpretation of behavioural experiments [32,33]. By thoroughly assessing and reporting the sensory information available to the animal (in this case, colour profiles of the illuminating light and the targets used), researchers can provide valuable data to accurately interpret their results. Such colour profiles can easily be measured using available technology (e.g. spectrophotometers). Unfortunately, this is not commonplace.

**Figure 3.**
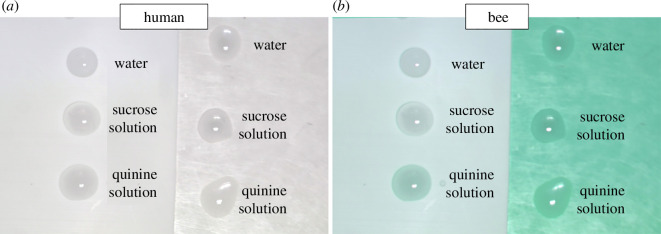
Drops of distilled water, sucrose solution and quinine solution appear identical to humans (*a*), but to bees, quinine solution is subtly tinted, owing to its ultraviolet-absorbing properties (*b*). This effect will, however, depend on whether the drops are presented on an ultraviolet-reflective (left) or ultraviolet-absorbent (right) background. The false-coloured image of bee vision was made using Vasas *et al*. [17]. The animal’s ultraviolet, blue, and green colour channels are shown as red, blue and green, respectively. Pictures were taken in an indoor laboratory setting, under a broadband light whose emission spectrum included ultraviolet (for details, see [17]). A gamma correction of 0.5 was applied to the images.

Let’s consider, for instance, the question of quinine solution, commonly used in behavioural experiments with bumblebees and honeybees. Quinine solution is distasteful to bees, therefore it is often used as a punishment [34,35]: the bee strives to avoid the quinine and quickly learns to associate it with the incorrect choice in the behavioural task. This design relies on the underlying assumption that the bee perceives the quinine–water solution as a transparent and odourless liquid. The well-established fact that quinine solution absorbs ultraviolet light and fluoresces is typically overlooked [36] (S1, E1, M1, table 1). Presumably, an animal with ultraviolet-sensitive photoreceptors and excellent colour vision, such as bees, should be able to perceive its colour (figure 3). Yet, from 22 recent papers that used quinine as punishment in visual learning tasks (published between Jan 2019 and Aug 2023 identified by searching for the keywords ‘quinine+bee’ on Google Scholar [35,37–57]), none mentioned this potential confounding factor.

Complicating this picture further, control experiments found that bees did not learn to avoid targets identified by a drop of quinine solution [58,59]. This is confusing, as other experiments show that bees readily use UV signals when foraging [31,60,61]. There is a possibility that in the control experiments the drops were presented on ultraviolet-absorbent background, and the colour of this background masked the subtle colour of quinine solution (figure 3). This explanation still leaves open the possibility that in other experiments where the drops are presented on ultraviolet-reflective backgrounds, the bees could avoid punishment by identifying quinine solution by its colour. For this reason, it would be ideal if each separate experiment included controls for quinine (as in Dyer & Neumeyer and Whitney *et al*. [58,59]; M1, M2, table 1). Unfortunately, if the colour of quinine influenced the 22 recent articles, we cannot confirm it retrospectively. Only two papers [41,53] contain the necessary information on both ultraviolet illumination and the ultraviolet-reflectivity of the background upon which the quinine solution drop was placed. In a further four papers, the solutions were presented in small plastic tubes or in holes drilled into small cubes and balls [47,48,50,56], and were, therefore, most likely hidden from bees. In most cases, however, the droplets were presented on landing platforms or poles whose ultraviolet reflectance is not described, or the papers do not specify whether ultraviolet illumination was present [35,37–40,42–46,49,51,52,54,55,57]. On top of these questions around the colour of quinine solution, some evidence has even suggested bees can visually distinguish sucrose solution (commonly used as a reward) from water [62].

If bees indeed discriminate between the solutions used during training, how would that affect experimental outcomes? The answer is not straightforward and depends heavily on the experimental methods. False positives may occur if sucrose and quinine solutions are available during testing phases. This would overinflate our interpretation of bees’ abilities. False negatives may occur if sucrose and quinine are *not* available during testing phases after bees were exposed to these solutions during training. To the bee, a sudden visual change at the testing phase could disrupt a normal learnt behaviour or suggest that no reward is available. It is also possible that bees could benefit from the extra visual cues, the same way they do from multiple, synergistic cues, and learn the trained stimuli faster when sucrose and quinine solutions are visible [63]. Whether any biases are relevant and how they will alter the experimental outcome is hard to predict, especially within this current lack of reported UV information (S1, E1, table 1).

Nevertheless, for an animal with well-established visual learning abilities, more emphasis on the visual environment (as trivial as reporting lighting conditions) can only improve our interpretations. Recent evidence highlights the fundamental effect of visual cues on observed behaviour. In one experiment, when bumblebees were tested in a complex cognitive task, they succeeded or failed according to the colours used during training [64]. In another study, Australia’s native bees were found to prefer native flowers in the field, but chose non-native flowers in the laboratory [65]. What appears to be clear is that we need to push for more comprehensive reporting of the relevant environmental cues (E1, table 1).

## 3. Consider new confounds in new set-ups: research animals living in the digital age

One particular area in which careful consideration must be taken is in the use of electronic displays. Monitors and similar methods to display stimuli have been gaining popularity owing to their ease of use; however, they are deceptively difficult to tailor to the vision of non-human species (M1, table 1). First of all, the three primary colours of monitors have been carefully designed to recreate human-perceived colours of objects, but how they appear to species with different visual systems has not been experimentally tested. Second, a standard desktop monitor has a refresh rate of 60 Hz, which is too fast for humans to detect but appears to flash to a variety of animals that have a faster flicker fusion frequency (the rate at which a flicker becomes invisible) [66,67]. A honeybee would even detect the flashing screen updates of professional gaming monitors, whose refresh rates of 120–144 Hz fall below the honeybee eyes’ fusion frequency of 200 Hz [68]. This is even without trying to recreate fluid motion on a monitor, which for humans requires a much higher refresh rate (e.g. even at 120–144 Hz, we still perceive multiple frames instead of fast movement when, for example the mouse on screen is moved quickly). For animals with a higher flicker fusion frequency, smooth fast motion would then require even higher refresh rates [69–71]. For these reasons, using a monitor with refresh rates adapted to the flicker fusion frequency of the species and using still or slow-moving stimuli is a good first step to avoiding possible confounds.

Even beyond the problems associated with colours and flicker, monitor displays deviate from natural targets in multiple ways: as well as presenting a two-dimensional display that is problematic for representing three-dimensional objects, some emit polarized light, heat or generate an electrostatic field, all of which are perceivable to many insects [72–75]. Being unaware of these issues could easily lead to flawed designs or faulty interpretations. For example, dogs can be trained to attend to visual stimuli on a monitor screen for up to 10 s, and can distinguish separate movement patterns in this set-up [76]. However, when the authors repeated the same experiment with remote-controlled toy cars, the dogs paid attention to the demonstration substantially longer and spontaneously, without the need for training [77]. Even more importantly, the dogs demonstrated a clear preference for the cars that appeared to have moved purposefully, an effect that was significantly dampened when they viewed moving objects on screens [77]. Similarly, zebrafish showed a robust and consistent fear response when exposed to a robotic replica of their predator, but not when confronted with a computer-generated animation [78]. On the other hand, stimuli displayed on monitors have elicited naturalistic hunting and display behaviour in jumping spiders [79,80], prey capture in praying mantises [81], imprinting (of colours, shapes, etc.) and spontaneous approach responses in domestic chicks [82,83] and elicited pecking in (trained) blue jays [84]. Why monitors do not always elicit the same responses as physical stimuli is unclear. Considering the multiple unknowns associated with screens, it becomes essential to consider whether using a monitor is necessary and report the cues that may affect the experiments (such as refresh rate and primary colours of the monitor, frame-per-second rate if using a video, etc.). The success or failure of each case depends on the experimental context as much as on the animal’s sensory system; therefore, the possible consequences of using electronic displays—like any novel set-up—needs to be assessed on a case-by-case basis (M1, table 1).

## 4. Consider how your species responds to sensory information: using the right language to ask questions

We know far more about how animals’ sensory organs receive signals than we do about how they process those signals. Yet, organisms process and interpret sensory information in species-specific ways that are not necessarily intuitive to us, which poses an increased risk of relying on anthropocentric biases. Again, the deviations from the anthropocentric expectation tend to be stronger in more distantly related species. For example, inexperienced mosquito-eating jumping spiders (*Evarcha culicivora*) are just as likely to pounce on disassembled stick figure representations of mosquitoes (strikingly different to a human eye) as assembled stick-figure mosquitoes, as long as the relative angles of the elements stay the same (analogous to a scenario where the ‘legs’ are pointing in the right direction) [85]. Another example is the stereopsis (depth perception) of praying mantises, which appears superficially similar to ours, but operates on different principles and as a result allows them to detect depth cues of camouflaged moving targets with a higher accuracy and in different conditions than human observers [81]. Even in vertebrates, studies on animal responses to visual illusions found that other species can respond to the same stimulus in opposing ways, indicating that they processed and interpreted the scene differently (e.g. Delboeuf illusion in guppies versus humans [86,87]). These processing differences extend into our understanding of the behaviour of interest itself, such as whether it is learnt and what constitutes its triggers (S2, M2, table 1).

Beyond the above issues specific to animal vision, the sensory domain of the experimental design may itself be less than optimal. Humans primarily rely on vision, and as a result tend to design experiments using visual cues. Consider, for example the mirror test of recognition. The test was originally designed for identifying animals that possess a concept of the self and led to the suggestion that self-recognition might be limited to great apes [88,89]. Even species with well-understood intelligence (e.g. New Caledonian crows [90]) failed the mirror test. However, vision is not the primary source of information for many organisms who might use a different sensory domain to recognize each other or themselves [91–93]. Accordingly, it has been suggested that dogs might pass a modified ‘olfactory mirror test’ using smell [93]. There are also instances of raptors, known for mainly relying on vision, preferring olfactory information in certain contexts [94]. Beyond olfaction, many commonly studied insects (ants, bees, locusts, flies) are sensitive to electrostatic gradients [73,95,96], airflow and vibratory information [97], and to magnetic cues [98–100], and yet these sensory domains are not routinely used as cues in experiments carried out with them. In these cases, the sensory domain of the presentation itself will pose an additional challenge for the animal and may explain poor performance.

## 5. Assume sensory ability exists if there is no evidence to disprove it: the case of the smelling birds

Sometimes, experiments fall victim to a different kind of error: ignoring an important sensory domain altogether. When studying other species, there is of course the chance that they can detect cues we do not know to look for. However, when we can identify possible sensory cues, it is essential that these are treated as possible confounds (S1, E1, M1, table 1). This may seem obvious, but researchers have fallen into the trap of ignoring entire sensory modalities without proof owing to widespread false beliefs (e.g. magnetosensation, highlighted in Nimpf & Keays [101]). In some cases, the incorrect assumptions about animal sensory systems prevail unnecessarily long. For example, despite strong evidence suggesting the potential importance of olfaction in birds dating back to 1911 [102], the misconception that most birds have poor olfactory abilities has been lingering in research and textbooks for decades [103–106]. These types of misconceptions can introduce important confounds to otherwise rigorous experiments (S1, S2, E1, M2, table 1).

As it becomes increasingly clear that birds can use olfaction in many contexts [107–111], including lab-based two-choice trials [109], egg recognition in the wild [110] and in mate choice [112–114], an increasing number of avian studies are regarding olfaction as a possible confound. Consider, for instance, experiments focusing on discrimination tasks that ask subjects to choose between two or more stimuli to find a hidden treat. These designs can provide powerful insight into cognition, through their ability to study choice, association, and inference [115–117]. Naturally, if the subject can use olfaction to detect the hidden food item, the task can be solved without inference. Luckily, experiments on birds can easily control for the confounding factor of smell through applying appropriate controls (M1, M2, table 1). For example, the researcher can provide incomplete (or no) visual information to determine if the treats can still be reliably located using scent [118–121]. Danel *et al*. [122] applied a strong scent of food on both cups, attempting to cover and hide existing olfactory information. These more recent experiments are only the start of a growing body of literature that is carefully considering the sensory systems of non-human animals.

## 6. Consider species’ lifestyle, lifecycles and ecology: the early bird finds a tired lab rat

Finally, ecology and habitat are important aspects of an animal’s behaviour and should be considered when designing experiments (E1–E3, table 1) [123]. Rats and mice have been used widely and for over a century in psychological and behavioural research, but only recently did a few experimenters account for the fact that these rodents are nocturnal and started testing them in darkness (in practice, under red light that is not visible to them [124,125]; E3, table 1). The time of day at which rodents are tested is still often unreported or inappropriate to the species’ known active periods even in highly cited research [126]. These issues exacerbate the already perilous practice of extrapolating from model animals to humans in medical research [127,128]. Beyond this, several studies [129,130] have emphasized that the acoustic environment alters the behaviour and welfare of laboratory animals (E2, table 1). Governmental guidelines and regulations recommend monitoring noise levels across a broad wavelength range, including ultrasonic noise (over 20 kHz), which is produced by many common laboratory fittings, including dripping taps, trolley wheels and computer monitors [131]. However, there is a lack of information on the acceptable levels of ultrasound noise and the tools that should be used for measuring it [132]. Moreover, the regulations do not consider the type of light used for artificial illumination (whether it includes ultraviolet, and whether the 120 Hz flicker introduced by the AC electric systems is above the flicker fusion frequency of the animal). Sensory environments that are not designed with the animals’ needs in mind are likely to induce stress, alter behaviour and so lead to poor performance in cognitively demanding tasks (E1, E2, table 1). Again, animals may fail tests for high cognitive abilities not because they lack them but because of constraints posed by a human-centred testing approach.

## 7. Conclusion

Experimental design (in the broad sense) can profoundly alter the outcome of scientific work. It can also be a complex challenge, especially when designing for another species’ perception. The stakes are higher than ever: getting an accurate picture of the abilities and minds of animals is crucial for a wide range of topics, from medical insights [1,133], ecology [134–137] and conservation [138], to the recent debate on animal sentience [139–141]. All these fields draw evidence from behavioural experiments and observations, which in turn are strongly impacted by each animals’ sensory abilities.

We need to remember that animals perceive the world differently to us, and that these differences directly influence experimental outcomes. We need to ask ourselves: how does an animal perceive the world? What can it see, smell, taste? What senses does it possess that we do not? Although these questions are not new, we argue that they need to be reiterated and directly addressed (table 1). Anthropocentric design flaws appear in our experiments despite our best efforts. Our experimental toolsets enable remarkable scientific advances, but to detect subtle differences, we cannot afford to accept known confounding factors. For that reason, experimental paradigms need to be reassessed and regrounded in the sensory biology of each species. We have the necessary tools: a wealth of information on sensory biology, appropriate measurement techniques and species-specific models to estimate the sensory information available for our experimental animals. Only by making full use of these tools and incorporating our test subjects’ perspective can we achieve a fair assessment of the abilities of animal minds.

## Data Availability

This article has no additional data.
